# Circulating miRNA-3552 as a Potential Biomarker for Ischemic Stroke in Rats

**DOI:** 10.1155/2020/4501393

**Published:** 2020-07-16

**Authors:** Litao Li, Lipeng Dong, Jingru Zhao, Weiliang He, Bao Chu, Jijie Zhang, Zongkai Wu, Congying Zhao, Jinming Cheng, Wentao Yao, Hebo Wang

**Affiliations:** Department of Neurology, Hebei General Hospital, No. 348, Heping West Road, Shijiazhuang, 050050 Hebei Province, China

## Abstract

**Objective:**

With the growing incidence of ischemic stroke worldwide, there is an urgent need to identify blood biomarkers for ischemic stroke patients. Thus, our aim was to identify potential circulating microRNA (miRNA) as a potential biomarker and to explore its potential mechanism for ischemic stroke in rats.

**Methods:**

The mRNA dataset GSE97537 and miRNA dataset GSE97532 were downloaded from the Gene Expression Omnibus (GEO) GSE97537 including 7 middle cerebral artery occlusion (MCAO) rat brain tissues and 5 sham-operated rat brain tissues GSE97532 including 6 MCAO rat blood samples and 3 sham-operated rat blood samples. Differentially expressed mRNAs and miRNAs with corrected *p* value ≤ 0.01 and fold change ≥2 or ≤0.05 were identified. To explore potential biological processes and pathways of differentially expressed mRNAs, functional enrichment analysis was performed. The target mRNAs of differentially expressed miRNAs were predicted using DNA Intelligent Analysis (DIANA)-microT tools. The target mRNAs and differentially expressed mRNAs were intersected.

**Results:**

1228 differentially expressed mRNAs in MCAO rat brain tissues were identified. Highly expressed mRNAs were mainly enriched in the inflammatory responses. Nine differentially expressed miRNAs were identified in MCAO rat blood samples. A total of 673 target mRNAs were predicted to significantly bind these differentially expressed miRNAs. Among them, 54 target mRNAs were differentially expressed in MCAO rat blood samples. Enrichment analysis results showed that these 54 target mRNAs were closely related to neurological diseases and immune responses. Among all miRNA-mRNA relationship, miR-3552-CASP3 interaction was identified, indicating that CASP3 might be mediated by miR-3552. Functional enrichment analysis revealed that CASP3 was involved in the apoptosis pathway, indicating that miR-3552 might participate in apoptosis by CASP3.

**Conclusion:**

Our findings reveal that circulating miR-3552 shows promise as a potential biomarker for ischemic stroke in rats.

## 1. Introduction

Stroke is the third leading cause of death after heart disease and cancer, classified as ischemic (85%) and hemorrhagic (15%) [1]. The world causes more than 6 million deaths each year. Ischemic stroke occurs when cerebral blood flow is interrupted, usually due to arterial thrombosis or embolism. In addition to ischemia, reperfusion also impairs the brain after ischemia, including inflammation and oxidative stress. Ischemic stroke poses a major threat to patient health and quality of life. However, the pathogenic mechanisms of ischemic stroke remain to be clarified, leading to an ideal clinical treatment [2, 3]. Therefore, understanding the molecular neuroinflammatory mechanism of ischemic stroke can significantly enhance the development of treatment. Prognostic blood biomarkers provide a novel strategy for the diagnosis and prognosis of stroke [4]. Currently, the diagnosis of ischemic stroke usually relies on computed tomography. The diagnostic value of blood biomarkers in ischemic stroke remains limited.

MicroRNAs (miRNAs), a class of noncoding RNAs, play an essential role in neurological diseases, including ischemic stroke [5]. Increasing evidence demonstrates that miRNAs participate in various biological processes, such as proliferation, apoptosis, and inflammatory response [6–9]. MiRNAs may regulate gene expression at the posttranscriptional level by binding 3′-UTR of target mRNAs [10, 11]. Due to the stability of miRNAs in peripheral blood and specific expression patterns in cell types, miRNAs show promise as diagnostic and prognostic markers of ischemic stroke [12]. Ischemic stroke may affect the expression levels of circulating miRNAs through various pathophysiological processes such as immune and inflammatory responses [13]. Therefore, it is necessary to explore the pathophysiological processes involved in miRNAs. It has been reported that serum miRNA-mRNA interactions play critical roles in the progression of ischemic stroke [14]. High-throughput sequencing technology of miRNAs and mRNAs for ischemic stroke both in animal models and Homo sapiens has been conducted [15–17]. However, the relationships between miRNAs and mRNAs remain to be clarified.

GEO, an online public database, is provided by the National Center for Biotechnology Information in 2000, which has become one of the most comprehensive gene expression data resources available. Based on this database, we performed a comprehensive analysis for gene expression pattern both in the MCAO rat blood and tissues samples that have been widely used as classical models for stroke.

## 2. Materials and Methods

### 2.1. Expression Data and Data Preprocessing

The mRNA dataset GSE97537 and miRNA dataset GSE97532 [18] were retrieved from the GEO (https://www.ncbi.nlm.nih.gov/geo) database. GSE97537 was generated on GPL1355 [Rat230_2] Affymetrix Rat Genome 230 2.0 Array platform. A total of 7 brain samples from MCAO rats and 5 brain samples from sham-operated rats were included in the GSE97537 dataset. GSE97532 was on the basis of GPL21572 [miRNA-4] Affymetrix Multispecies miRNA-4 Array [ProbeSet ID version] platform. Totally, of 6 blood samples from MCAO rats and 3 blood samples from sham-operated rats were included in the GSE97532 dataset.

Expression data of probes were annotated as corresponding gene or miRNA symbols on the basis of GPL1355 or GPL21572 platform, respectively. For one gene or miRNA corresponding to multiprobes, the maximum expression value was considered as the expression value of this gene or miRNA. After that, the expression matrix was constructed. For GSE97537 dataset, there were a total of 31099 probes in each sample (Supplementary Table 1). After preprocessing, 15239 genes were obtained. A total of 1277 miRNAs were included in GSE97532 dataset (Supplementary Table 2). Following preprocessing, 541 miRNAs were retained for further analysis.

### 2.2. Differential Expression Analysis

The differentially expressed mRNAs between 7 MCAO rat brain tissues and 5 sham-operated rat brain tissues, and miRNAs between 6 MCAO rat blood samples and 3 sham-operated rat blood samples were analyzed by Linear Models for Microarray Data (limma) package (version 3.34.7; https://bioconductor.org/packages/release/bioc/html/limma.html), respectively. Then, fold change and corrected *p* value were generated by the limma package [19]. The corrected *p* value ≤ 0.01 and log2|fold change| ≥2 or ≤0.05 were set as thresholds.

### 2.3. Functional Enrichment Analysis

Gene set enrichment analysis (GSEA: http://software.broadinstitute.org/gsea/index.jsp) was performed to explore potential functions associated differentially expressed mRNAs [20]. *p* value = 0.01, *q* value = 0.01, and Jacard >0.375 were set as the cut-off criterion. Gene ontology (GO) database was used to annotate gene functions [21]. GO function terms include molecular function (MF), biological process (BP), and cellular component (CC). Furthermore, Kyoto Encyclopedia of Genes and Genomes (KEGG) pathway analysis [22] was performed using the Database for Annotation, Visualization and Integrated Discovery (DAVID; version 6.8; (https://david.ncifcrf.gov/)) [23]. *p* value < 0.05 was considered to be significantly enriched.

### 2.4. RT-qPCR Assay

14 rats were purchased from Shanghai Experimental Animal Center (Shanghai, China). These rats were randomly separated into MCAO (*n* = 11) and sham operation (*n* = 3). The MCAO model was conducted, as previously described [18]. 24 hours after the reperfusion, the rats were anesthetized. The blood and brain tissue samples were collected. Total RNA was extracted using Trizol (solarbio, Beijing, China), which was reverse transcribed into cDNA utilizing reverse transcription kit (YEASEN, Shanghai, China). RT-qPCR was carried out by qPCR kit (YEASEN). The relative expression levels were determined with the 2^–*ΔΔ*Ct^ method. Our research was approved by the Ethics Committee of Hebei General Hospital.

### 2.5. miRNA Target Prediction

The targets of differentially expressed miRNAs were predicted using DIANA-microT tools that identify mRNA targets for mouse miRNAs [24]. Furthermore, the targeted mRNAs of the interested miRNA was predicted using TargetScan [25], miRTarBase [26], miRDB [27], miRanda [28], and miRMap [29] databases. The miRNA-mRNA interaction network was visualized using Cytoscape software [30].

## 3. Results

### 3.1. Identification of Differentially Expressed mRNAs between MCAO Rat Brain Tissues and Sham-Operated Rat Brain Tissues

**Figure 1:**
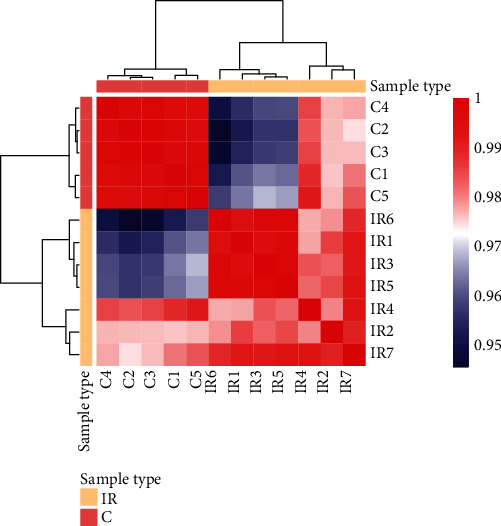
Sample hierarchical clustering analysis. Based on the gene expression matrix, the correlation coefficients between samples were calculated. After construction of the correlation coefficient matrix, the Euclidean distance of the correlation coefficients between the samples was obtained to conduct the heat map.

The similarity test of the samples was carried out to determine whether there was a difference in similarity between the samples at the expression level. Using the correlation coefficient matrix, we found the differences in transcription levels between 7 MCAO rat brain tissues and 5 sham-operated rat brain tissues (Figure 1). Therefore, further downstream analysis can be performed.

**Figure 2:**
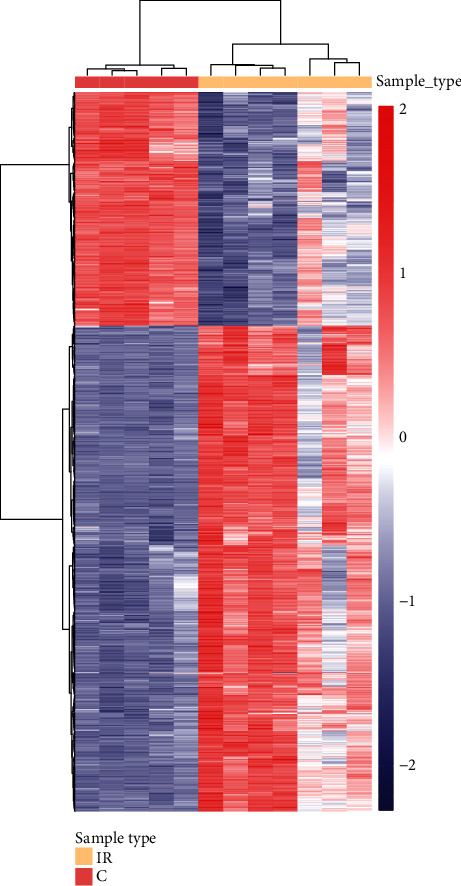
Hierarchical clustering analysis of differentially expressed mRNAs. The differentially expressed mRNAs were used to construct expression matrix. Data was normalized by scores. The samples and mRNAs were clustered on the basis of the Euclidean distance.

We identified 1228 differentially expressed mRNAs with corrected *p* value ≤ 0.01 and fold change ≥2 or ≤0.05 between MCAO rat brain tissues and sham-operated rat brain tissues, including 829 up-regulated and 399 down-regulated mRNAs (Figure 2, Supplementary Table 3).

### 3.2. Gene Enrichment and Functional Annotation of Differentially Expressed mRNAs

**Figure 3:**
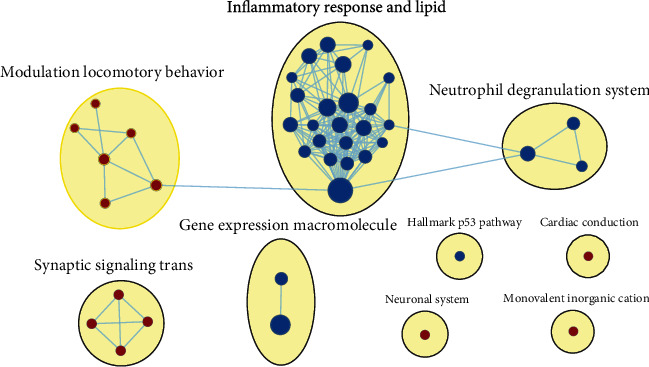
Gene enrichment and functional annotation of differentially expressed mRNAs. Blue represents a highly expressed mRNA in MCAO rat brain tissues, and red represents a highly expressed mRNA in sham-operated rat brain tissues.

After obtaining differentially expressed mRNAs, we first used GSEA software to perform enrichment analysis on differentially expressed mRNAs to elucidate their functions. Herein, the results with *p* value = 0.01, *q* value = 0.01, and Jacard >0.375 were considered to be significantly enriched. The similar gene functions were annotated on the basis of the GO database. We found that among the differentially expressed mRNAs, highly expressed mRNAs in MCAO rat brain tissues were mainly enriched in the inflammatory responses, as shown in Figure 3. The top ten gene enrichment and functional annotation results of differentially expressed mRNAs are listed in Supplementary Table 4.

### 3.3. Pathway Enrichment Analysis of Differentially Expressed mRNAs

**Figure 4:**
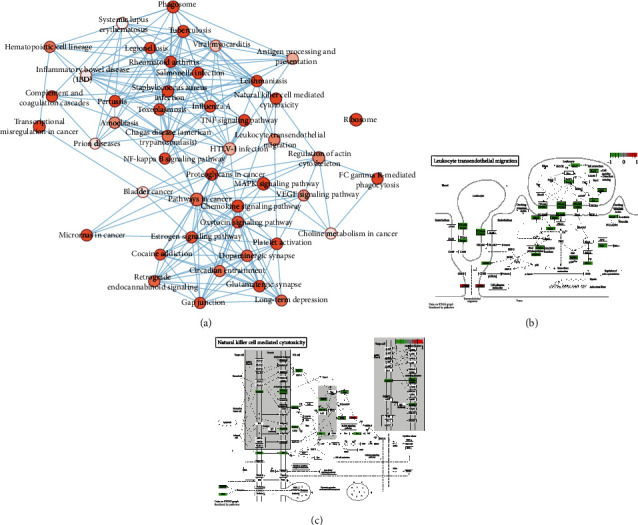
Pathway enrichment analysis of differentially expressed mRNAs. (a) 85 pathways enriched by 1288 differentially expressed mRNAs. The circle represents the signal pathway. The red shade in the circle represents the *p* value, the deeper the color, the smaller the *p* value. (b) The differentially expressed mRNAs in leukocyte transendothelial migration pathway. Green represents highly expressed mRNA in MCAO rat brain tissues, and red represents lowly expressed mRNA in MCAO rat brain tissues. (c) Differentially expressed mRNAs in natural killer cell-mediated cytotoxicity pathway. Green or red stands for highly or lowly expressed mRNA in MCAO rat brain tissues, respectively.

Based on gene enrichment analysis, we performed pathway enrichment analysis to identify functional features of differentially expressed mRNAs. The online KEGG pathway enrichment analysis tool DAVID was used for pathway enrichment analysis. The results showed that 1288 differentially expressed mRNAs were enriched into 85 pathways. Among them, genes that were highly expressed in MCAO rat brain tissues were mainly enriched in pathways related to immune and inflammatory responses (Figure 4(a)). Among all enriched pathways, leukocyte transendothelial migration and natural killer cell-mediated cytotoxicity were significantly enriched. The differentially expressed mRNAs in leukocyte transendothelial migration and natural killer cell-mediated cytotoxicity pathways were, respectively, shown in Figures 4(b) and 4(c).

We further analyzed whether these enriched pathways were expressed in MCAO rat brain tissues. The results showed that 26 signaling pathways were significantly positive in MCAO rat brain tissues (Table 1), and 6 pathways were significantly negative in MCAO rat brain tissues (Supplementary Table 5).

### 3.4. Differentially Expressed miRNAs between MCAO Rat Blood Samples and Sham-Operated Rat Blood Samples

Nine differentially expressed miRNAs were identified between MCAO rat blood samples and sham-operated rat blood samples (Table 2), including rno-mir-191b, rno-mir-743a, rno-miR-128-2-5p, rno-miR-383-5p, rno-miR-3552, rno-miR-107-5p, rno-miR-137-3p, rno-mir-194-1, and rno-mir-429.

### 3.5. miRNA Target Prediction

After identification of differentially expressed miRNAs, target genes of differentially express miRNAs were predicted using DIANA Tools. A total of 673 genes were predicted to significantly bind these differentially expressed miRNAs (Supplementary Table 6).

### 3.6. Intersection of Differentially Expressed mRNAs and Predicted Targets of Differentially Expressed miRNAs

**Figure 5:**
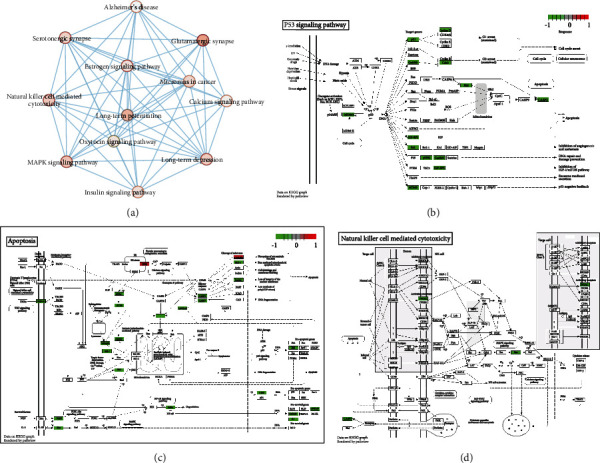
Pathway enrichment analysis of differentially expressed mRNAs targeted by differentially expressed miRNAs. (a) Signaling pathways enriched by differentially expressed mRNAs targeted by differentially expressed miRNAs. (b) Differentially expressed mRNAs in p53 signaling pathway. (c) Differentially expressed mRNAs in apoptosis pathway. (d) Differentially expressed mRNAs in natural killer cell-mediated cytotoxicity pathway. Green or red stands for highly or lowly expressed mRNA in MCAO rat brain tissues, respectively.

Differentially expressed mRNAs and predicted targets of differentially expressed miRNAs were overlapped. We found that 54 out of target genes of differentially expressed miRNAs were differentially expressed in MCAO rat blood samples. Enrichment analysis results showed that these 54 target mRNAs were closely related to neurological diseases and immune responses (Figure 5(a)).

Notably, we found that CASP3 was significantly highly expressed in MCAO rat brain tissues, while rno-miR-3552 was significantly lowly expressed in MCAO rat blood samples. It was predicted that CASP3 was one of the target genes of miR-3552. Furthermore, CASP3 was involved in the apoptosis pathway, as shown in Figures 5(b)–5(d).

### 3.7. Validation of rno-miR-3552 and Prediction of Its Targeted mRNAs

**Figure 6:**
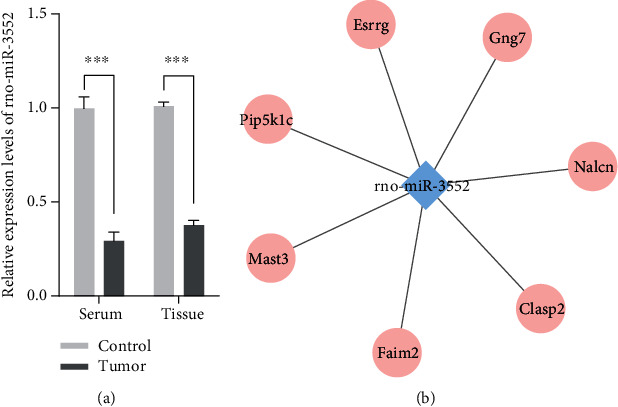
Validation of rno-miR-3552 and prediction of its targeted mRNAs. (a) RT-qPCR was used to validate the expression of rno-miR-3552 both in blood and brain tissue samples of MCAO rats. (b) The targets of rno-miR-3552. Diamond represents rno-miR-3552, and circles represent the targeted mRNAs of rno-miR-3552. Red expresses up-regulation, and blue indicates down-regulation.

After validation using RT-qPCR, we found that rno-miR-3552 was significantly down-regulated both in blood and brain tissue samples of MCAO rats (Figure 6(a)). For the targeted mRNAs of rno-miR-3552, we searched TargetScan, miRTarBase, miRDB, miRanda, and miRMap databases to predict the corresponding miRNA-mRNA regulatory relationship pairs. Cytoscape [6] software was to construct miRNA-mRNA regulatory network. The results are shown in Figure 6(b). We found that Esrrg, Gng7, Nalcn, Clasp2, Faim2, Mast3, and Pip5k1c could be potential targets of rno-miR-3552.

### 3.8. Functional Enrichment Analysis of Targeted mRNAs Regulated by rno-miR-3552

**Figure 7:**
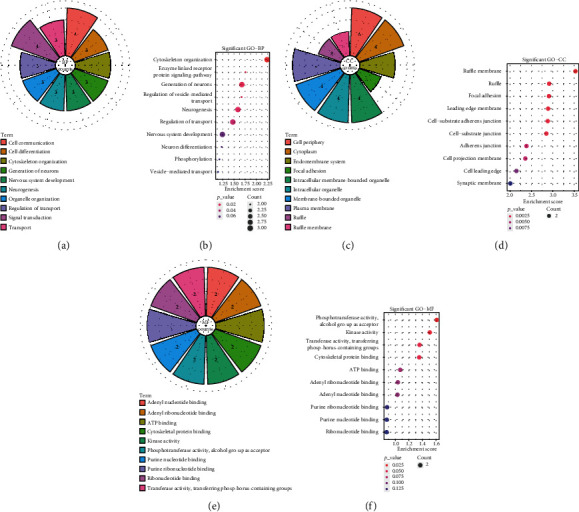
GO annotation analysis results showing potential functions of targeted mRNAs regulated by rno-miR-3552. (a, b) Biological processes; (c, d) cellular component; (e, f) molecular function.

**Figure 8:**
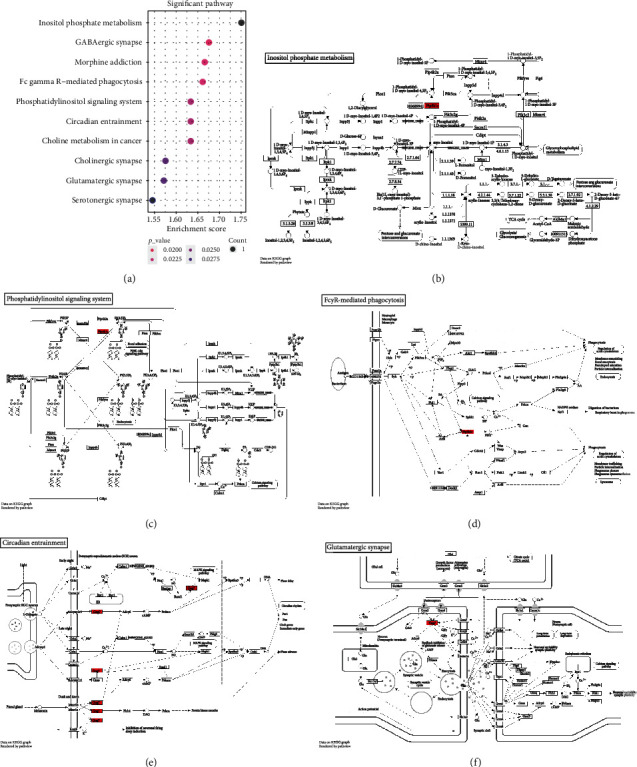
KEGG pathways enriched by targeted mRNAs of rno-miR-3552. (a) The top ten KEGG pathways. (b) Inositol phosphate metabolism; (c) phosphatidylinositol signaling system; (d) Fc gamma R-mediated phagocytosis; (e) circadian entrainment; (f) glutamatergic synapse.

**Figure 9:**
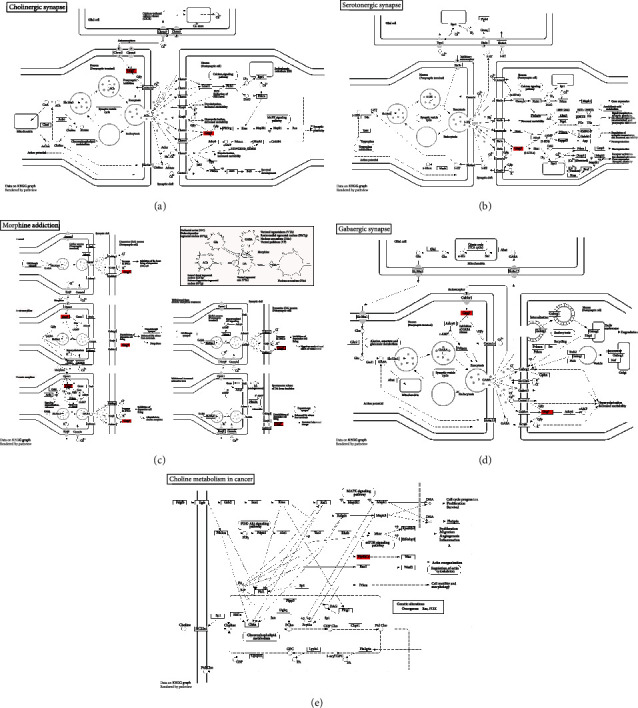
KEGG pathways enriched by targeted mRNAs of rno-miR-3552. (a) cholinergic synapse; (b) serotonergic synapse; (c) morphine addiction; (d) GABAergic synapse; (e) choline metabolism in cancer.

To explore the function of targeted mRNAs regulated by rno-miR-3552, functional enrichment analysis was presented. GO annotation analysis results showed the top ten BP (Figures 7(a) and 7(b)), CC (Figures 7(c) and 7(d)), and MF (Figures 7(e) and 7(f)). The details are listed in Supplementary Table 7. The top ten KEGG pathway analysis results (Figure 8(a)) are as follows: inositol phosphate metabolism (Figure 8(b)), phosphatidylinositol signaling system (Figure 8(c)), Fc gamma R-mediated phagocytosis (Figure 8(d)), circadian entrainment (Figure 8(e)), glutamatergic synapse (Figure 8(f)), cholinergic synapse (Figure 9(a)), serotonergic synapse (Figure 9(b)), morphine addiction (Figure 9(c)), GABAergic synapse (Figure 9(d)), and choline metabolism in cancer (Figure 9(e)).

## 4. Discussion

In this study, to improve the accuracy of results, we first confirmed the significant difference in similarity between MCAO rat brain tissues and sham-operated rat brain tissues. We identified 1228 differentially expressed mRNAs in MCAO rat brain tissues. The results showed that 829 highly expressed mRNAs were mainly enriched in inflammatory response. More importantly, pathway enrichment analysis results showed that inflammatory response such as leukocyte transendothelial migration and natural killer cell-mediated cytotoxicity was significantly activated in MCAO rat brain tissues. It has been confirmed that inflammation is a key parameter of the pathophysiology of stroke [31, 32]. Especially in ischemic stroke, neuroinflammation leads to neurodegeneration and brain injury [33]. Therefore, our results indicated that these differentially expressed mRNAs could be involved in inflammatory response during ischemic stroke.

In this study, we identified 9 differentially expressed miRNAs in MCAO rat blood samples. Circulating miRNAs, as noninvasive biomarkers, show potential as potential biomarkers for the early detection of ischemic stroke [13, 34]. The miRNA target prediction results showed that 54 target mRNAs of differentially expressed miRNAs were differentially expressed in MCAO rat brain tissues. Among all miRNA-mRNA relationships, miR-3552-CASP3 interaction aroused our attention. Our results showed that miR-3552 was down-regulated in MCAO rat blood samples, and CASP3 was up-regulated in MCAO rat brain tissues. CASP3 has been confirmed as a blood biomarker for the early detection of stroke [35]. The results indicated that CASP3 could be regulated by miR-3552. Functional enrichment analysis revealed that CASP3 may be involved in the process of apoptosis. As a previous study, CASP3 is overactivated in ischemic stroke, leading to cell apoptosis [36]. Inhibiting CASP3 can reduce the volume of cerebral infarction and improve neurological deficits and edema [37]. Neuronal apoptosis is involved in the pathogenesis of ischemic stroke [38]. It has been confirmed that CASP3 is essential for the neuronal apoptosis through chromatin condensation and DNA fragmentization [39]. Increasing evidence suggests that CASP3 is a therapeutic target for ischemic stroke. For example, atorvastatin improves cognitive activity via CASP3 in ischemic stroke [40]. Our results indicated that miR-3552 could be involved in apoptosis via targeting CASP3 in ischemic stroke.

Since miRNAs could circulate in the body through blood vessels, they can regulate gene expression in various tissues, including brain [41–43]. The changes in CASP3 mRNA expression in the blood might be regulated by circulating miR-3552. During cerebral ischemia, the blood-brain barrier destruction occurs due to ischemic vascular endothelial dysfunction that is controlled by various signaling pathways [44, 45]. Although miRNAs are not translated into proteins, they can regulate the expression and function of protein-coding genes [46]. Our study found that CASP3 was highly expressed in brain tissue, which was consistent with previous studies. The dysregulation of miR-3552 expression could be closely related to the pathophysiology of the neurological dysfunction via CASP3. The regulatory relationship between miR-3552 and CASP3 suggests that miR-3552 in circulating blood can be used as a molecular marker of ischemic stroke. To further analyze the role of miR-3552 in ischemic stroke, its targeted mRNAs were predicted. Functional enrichment analysis revealed that these mRNAs were mainly enriched in several pathways related with ischemic stroke such as inositol phosphate metabolism [47] and phosphatidylinositol signaling system [48].

Our findings reveal that circulating miRNA-3552 shows promise as a potential biomarker for ischemic stroke in rats. Furthermore, miR-3552 could participate in the development of ischemic stroke through CASP3. However, further studies should be required to validate the miR-3552-CASP3 relationships in the ischemic stroke.

## 5. Conclusion

In this study, we comprehensively analyzed mRNA expression profile in MCAO rat brain tissues and miRNA expression profile in MCAO rat blood samples. Functional enrichment analysis revealed that differentially expressed mRNAs were mainly enriched in inflammatory response. The relationships among miRNA in blood and mRNA in brain tissues were predicted. CASP3 could be a target of circulating miR-3552, which provides a novel insight into the biological functions of miR-3552 as a potential biomarker for ischemic stroke.

## Figures and Tables

**Table 1:** Six significantly negative signaling pathways in MCAO rat brain tissues.

Pathways	*p* value	*q* value	Size
Calcium signaling pathway	0.00311675	0.1688712	22
Neuroactive ligand-receptor interaction	0.00837752	0.1688712	24
Pancreatic secretion	0.01135395	0.1688712	13
Vascular smooth muscle contraction	0.01165795	0.1688712	10
Salivary secretion	0.01431112	0.1688712	11
Long-term depression	0.02892722	0.2844509	13

**Table 2:** Nine differentially expressed miRNAs between MCAO rat blood samples and sham-operated rat blood samples.

miRNA	logFC	Average expression	*t*	Adjusted *p* value
rno-mir-191b	-0.667804	0.73125867	-4.2646122	0.002752
rno-mir-743a	0.629253	0.63185717	4.0304575	0.003796
rno-miR-128-2-5p	-0.6348807	1.05558633	-3.9963437	0.003981
rno-miR-383-5p	-0.8783367	3.81765167	-3.9650254	0.004159
rno-miR-3552	-0.5392907	0.640782	-3.7367906	0.005745
rno-miR-107-5p	-0.7065244	0.56472678	-3.6573127	0.006441
rno-miR-137-3p	0.52626167	0.5438935	3.6122412	0.006875
rno-mir-194-1	0.640289	0.6326905	3.59409531	0.007058
rno-mir-429	0.57579073	0.4865613	3.41499271	0.009174

## Data Availability

The data used to support the findings of this study are included within the supplementary information file(s).

## Abbreviations

GEO: Gene expression omnibus
MCAO: Middle cerebral artery occlusion
DIANA: DNA intelligent analysis
miRNAs: MicroRNAs
limma: Linear models for microarray data
GSEA: Gene set enrichment analysis
GO: Gene ontology
KEGG: Kyoto Encyclopedia of Genes and Genomes
DAVID: Database for Annotation, Visualization and Integrated Discovery.

## Supplementary Materials

Supplementary Materials Supplementary Table 1: a total of 31099 probes in GSE97537 dataset. Supplementary Table 2: a total of 1277 miRNAs in GSE97532 dataset. Supplementary Table 3: 1228 differentially expressed mRNAs between MCAO rat brain tissues and sham-operated rat brain tissues. Supplementary Table 4: the top ten gene enrichment and functional annotation results of differentially expressed mRNAs. Supplementary Table 5: 26 significantly positive signaling pathways in MCAO rat brain tissues. Supplementary Table 6: 673 target genes of differentially expressed miRNAs. Supplementary Table 7: the top ten functional enrichment analysis results of targeted mRNAs regulated by rno-miR-3552.
