# Supplementary material for: Circulating miRNA-3552 as a Potential Biomarker for Ischemic Stroke in Rats
Source: Biomed Res Int. 2020 Jul 16;2020:4501393. doi: 10.1155/2020/4501393 (PMC7381948; doi:10.1155/2020/4501393)
Supplement: Supplementary Materials — Supplementary Table 1: a total of 31099 probes in GSE97537 dataset. Supplementary Table 2: a total of 1277 miRNAs in GSE97532 dataset. Supplementary Table 3: 1228 differentially expressed mRNAs between MCAO rat brain tissues and sham-operated rat brain tissues. Supplementary Table 4: the top ten gene enrichment and functional annotation results of differentially expressed mRNAs. Supplementary Table 5: 26 significantly positive signaling pathways in MCAO rat brain tissues. Supplementary Table 6: 673 target genes of differentially expressed miRNAs. Supplementary Table 7: the top ten functional enrichment analysis results of targeted mRNAs regulated by rno-miR-3552. [file 4501393.f1.zip › Supplementary Materials.docx]

**Supplementary Materials:**

Supplementary table 1. A total of 31099 probes in GSE97537 dataset.

Supplementary table 2. A total of 1277 miRNAs in GSE97532 dataset.

Supplementary table 3. 1228 differentially expressed mRNAs between MCAO rat brain tissues and sham-operated rat brain tissues.

Supplementary Table 4. The top ten gene enrichment and functional annotation results of differentially expressed mRNAs.

Supplementary Table 5. 26 significantly positive signaling pathways in MCAO rat brain tissues.

Supplementary table 6. 673 target genes of differentially expressed miRNAs.

Supplementary table 7. The top ten functional enrichment analysis results of targeted mRNAs regulated by rno-miR-3552.
